# Effects of administration of ascorbic acid and low‐dose hydrocortisone after infusion of sublethal doses of lipopolysaccharide to horses

**DOI:** 10.1111/jvim.15896

**Published:** 2020-10-07

**Authors:** Melinda J. Anderson, Alina S. Ibrahim, Bruce R. Cooper, Andrew D. Woolcock, George E. Moore, Sandra D. Taylor

**Affiliations:** ^1^ Department of Basic Medical Sciences College of Veterinary Medicine, Purdue University West Lafayette Indiana USA; ^2^ Department of Veterinary Clinical Sciences College of Veterinary Medicine, Purdue University West Lafayette Indiana USA; ^3^ Bindley Bioscience Center Purdue University West Lafayette Indiana USA; ^4^ Department of Veterinary Administration College of Veterinary Medicine, Purdue University West Lafayette Indiana USA

**Keywords:** corticosteroid, endotoxemia, equine, sepsis

## Abstract

**Background:**

Sepsis is associated with ascorbic acid (AA) depletion and critical illness‐related corticosteroid insufficiency (CIRCI) in humans.

**Hypotheses:**

Intravenous infusion of lipopolysaccharide (LPS) would (a) decrease endogneous AA concentrations, (b) induce CIRCI and (c) administration of a combination of AA and hydrocortisone (HC) would have decreased indices of inflammation compared to either drug alone.

**Animals:**

Thirty‐two healthy horses.

**Methods:**

Randomized placebo‐controlled experimental trial. Horses were assigned to 1 of 4 groups (saline, AA and HC, AA only, or HC only). Treatments were administered 1 hour after completion of LPS infusion. Clinical signs, clinicopathological variables, pro‐inflammatory cytokine gene expression and production, and plasma AA concentrations were assessed at various time points. Serum cortisol concentrations and ACTH stimulation tests were used to detect CIRCI.

**Results:**

There was no effect of drug on clinical signs or pro‐inflammatory cytokine gene expression or production compared to controls at any time point. Administration of AA was associated with higher blood neutrophil counts 6 hours after LPS infusion (11.01 ± 1.02 K/μl) compared to other groups (8.99 ± 0.94 K/μL; *P* < .009). Adminstration of HC was associated with higher blood neutrophil counts 12 hours after LPS infusion (10.40 ± 0.75 K/μl) compared to other groups (6.88 ± 0.68 K/μl; *P* < .001). Serum cortisol increased from 5.11 ± 1.48 μg/dL before LPS administration to 9.59 ± 1.83 μg/dL 1 h after completion of LPS infusion (T1) without an effect of treatment (*P* = 0.59).

**Conclusions and Clinical Importance:**

Ascorbic acid and HC appeared to protect against LPS‐induced neutrophil depletion and could be considered as adjunctive therapy in horses with endotoxemia.

**Abbreviations**
β‐GUS: beta‐glucuronidase
AA: ascorbic acid
CIRCI: critical illness‐related corticosteroid insufficiency
GC: glucocorticoid
HC: hydrocortisone
HPLC‐MS/MS: high‐performance liquid chromatography mass spectrometry
IL‐1β: interleukin 1 beta
IL‐6: interleukin 6
iNOS: inducible nitric oxide synthase
LPS: lipopolysaccharide
MAP: mean arterial blood pressure
ROS: reactive oxygen species
SAA: serum amyloid A
SBA: serum biochemical analysis
SIRS: systemic inflammatory response syndrome
SVCT2: sodium‐dependent vitamin C transporter 2
TNF‐α: tumor necrosis factor alpha

## 1 INTRODUCTION

Sepsis is a leading cause of morbidity and death in both neonatal and adult horses.^1^, ^2^, ^3^, ^4^ Systemic inflammatory response syndrome (SIRS) is a component of sepsis and refers to a dysregulated inflammatory response to infection that can lead to rapid deterioration despite pathogen elimination.^5^ In horses, sepsis is often caused by gram‐negative bacterial infection and is associated with increases in inflammatory cytokine gene expression and acute phase proteins.^6^, ^7^, ^8^ Sublethal, low‐dose lipopolysaccharide (LPS; endotoxin) administered IV to horses results in moderate clinical signs and clinicopathological abnormalities typical of sepsis.^2^, ^9^, ^10^ This model of endotoxemia in horses increases pro‐inflammatory cytokine gene expression and production of reactive oxygen species (ROS).^10^, ^11^, ^12^, ^13^, ^14^ The stress response to critical illness, particularly increased cortisol, is important in immune regulation. Cortisol insufficiency during sepsis contributes to SIRS.^15^, ^16^, ^17^ Although increased serum cortisol is expected in septic horses, the increase can be inadequate for the degree of illness and is termed critical illness‐related corticosteroid insufficiency (CIRCI). This occurs in approximately 50% of septic neonatal foals and is diagnosed based on increased ACTH : cortisol ratio or a blunted cortisol response to ACTH administration.^18^, ^19^, ^20^, ^21^, ^22^ Similarly, adult horses with SIRS typically present with increased serum cortisol concentrations and increased ACTH : cortisol ratios.^23^

Ascorbic acid (AA) has potential for treating sepsis. Antioxidant effects of AA include scavenging of ROS and activation of other scavengers, including α‐tocopherol.^24^, ^25^, ^26^ Ascorbic acid inhibits enzymes that promote ROS production, such as inducible nitric oxide synthase (iNOS), thereby protecting endothelial integrity and mitigating cardiovascular collapse.^27^ In addition, AA is a cofactor for production of enzymes that synthesize vasopressin and catecholamines, which are important in maintaining tissue perfusion during sepsis.^28^, ^29^, ^30^ Ascorbic acid improves neutrophil chemotaxis and oxidative killing of bacteria, as well as T lymphocyte function in mice exposed to LPS.^31^, ^32^ Mice with AA deficiency have higher death rates from infection compared to those supplemented with AA.^31^, ^33^ Importantly, plasma concentrations of AA are reduced in septic patients due to consumption by inflammatory mediators.^34^, ^35^

Low‐dose hydrocortisone (HC) administration to septic human patients with CIRCI has not consistently improved outcomes.^36^, ^37^, ^38^, ^39^ This might be due to oxidative damage of glucocorticoid (GC) receptors, which prevents cellular uptake of cortisol or exogenous corticosteroids; treatment with AA reverses this damage.^40^, ^41^, ^42^ In addition, transport of AA into cells is mediated by sodium‐dependent vitamin C transporter‐2 (SVCT2), the expression of which is decreased in sepsis, and exogenous GC can upregulate its expression.^43^, ^44^ These synergistic effects suggest that combination therapy might be beneficial during sepsis. In fact, evidence is emerging that “metabolic resuscitation,” which includes treatment with AA and low‐dose HC, is beneficial in mitigating organ dysfunction, decreasing vasopressor requirements, and improving survival in septic people.^45^, ^46^

We hypothesized that (a) horses treated with AA and HC after IV LPS infusion would have decreased clinical and clinicopathological abnormalities, and decreased pro‐inflammatory cytokine gene expression and production, compared to those treated with either drug alone; (b) IV LPS infusion would induce CIRCI; and (c) IV LPS infusion would result in decreased endogenous AA concentrations.

## 2 MATERIALS AND METHODS

### 2.1 Animals and experimental design

A blinded, randomized placebo‐controlled experimental trial was performed in 32 healthy adult horses obtained from a university teaching herd. Horses were randomly assigned to 1 of 4 groups using a random number generator. Eight horses per group was expected to provide >80% power (*α* = .05) to show a significant difference between groups in neutrophil abnormalities 1 hour after LPS administration.^10^ For horses to be included in the study, they had to have a normal physical examination, including a normal pain score and normal mean arterial blood pressure (MAP), as well as a normal white blood cell (WBC) count and differential, serum biochemical analysis (SBA), and serum amyloid A (SAA).^47^, ^48^ In addition, all horses had to show a response to LPS infusion as determined by an increased rectal temperature (>101.5°F), an increased pain score (>13),^49^ or development of neutropenia within 2 hours of IV LPS administration during execution of the current study. A total of 40 horses were administered LPS, but 8 were excluded because of lack of response to LPS. Seventeen mares and 15 geldings (median age 15 years; range 3‐25 years) were included in the study. Eight breeds were represented, including 10 Quarter Horses, 8 Thoroughbreds, 6 Paint Horses, 4 Standardbreds, and one each of 4 other breeds. The median weight of the horses was 528 kg (range 400‐600 kg). Horses were housed individually in box stalls starting 1 day before the study day, and were returned to pasture the morning after the study day. All horses had free access to fresh water and grass hay. This study was approved by the university's Institutional Animal Care and Use Committee.

### 2.2 Endotoxin and drug administration

**FIGURE 1:**
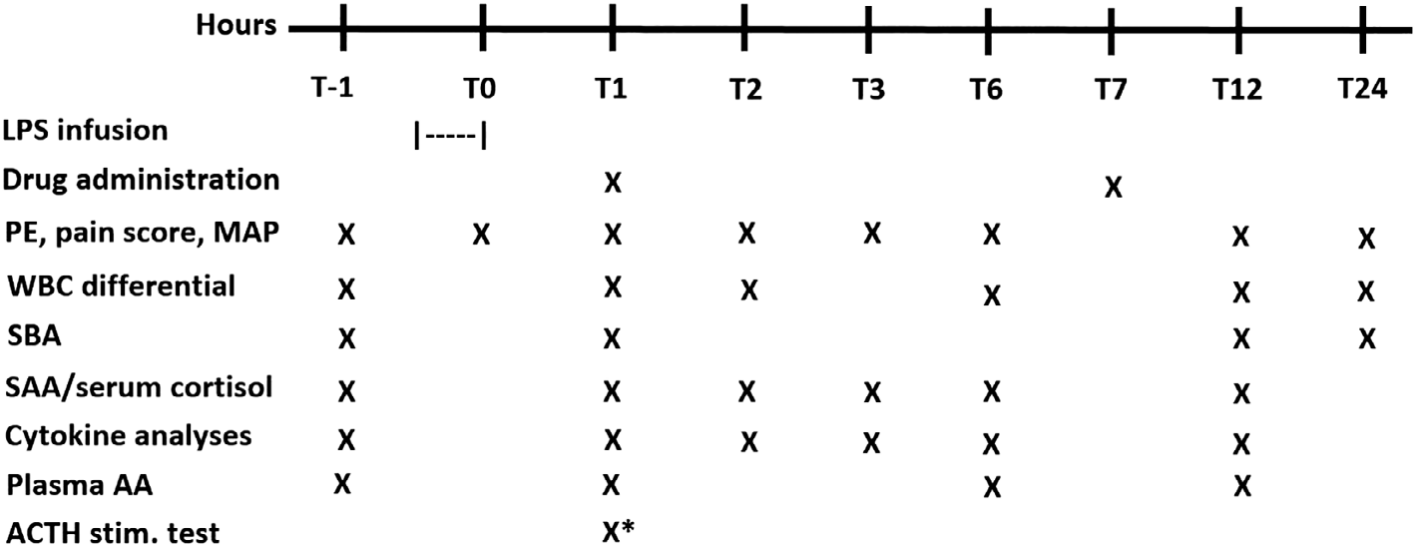
Daily schedule from T‐1h (baseline) to 24h post‐LPS infusion in 32 adult horses. The asterisk indicates that ACTH stimulation was performed only on horses administered 0.9% sodium chloride (placebo) and AA only. AA, ascorbic acid; ACTH, adrenocorticotropic hormone; LPS, lipopolysaccharide; MAP, mean arterial blood pressure; PE, physical examination; SAA, serum amyloid A; SBA, serum biochemical analysis; WBC, white blood cell

The study design and sampling protocol is shown in Figure 1. The evening before the start of the study, a jugular catheter was aseptically placed. Endotoxemia was induced in all horses by IV infusion of LPS (*Escherichia coli* 0111:B4) at 15 μg (0.025‐0.0375 μg/kg) in 500 mL 0.9% sodium chloride over a 30 minute period (T‐30min to T0h).^10^, ^13^, ^50^ Drug was administered 1 hour after completion of LPS infusion (T1) and to better simulate a clinical situation in which treatment would begin after LPS exposure. Drug was administered again 6 hours after the first dose (T7) based on the elimination half‐life of each drug and to mimic a reasonable dosing frequency in clinical cases.^51^, ^52^ The 4 treatment groups included 0.9% sodium chloride IV (placebo), AA and HC, AA only, and HC only. Horses in the placebo group were administered 30 mL of 0.9% sodium chloride IV as a bolus. This volume was similar to the volume of AA administered. Ascorbic acid was administered at 25 mg/kg IV as a bolus (Ascor, McGruff Pharmaceuticals, Santa Ana, California), the dose of which was based on pharmacokinetic studies in horses and extrapolation from human sepsis studies.^45^, ^51^ Hydrocortisone was administered at 0.3 mg/kg IV as a bolus (Solu‐Cortef, Pfizer, New York, New York). The dose of HC was based on ex vivo evidence of reduced pro‐inflammatory cytokine gene expression in peripheral blood mononuclear cells from HC‐treated foals after LPS exposure and modified for dosing frequency.^53^ In the combination group (AA + HC), the same doses of AA and HC were administered as in the individual drug administration groups (25 mg/kg IV and 0.3 mg/kg IV, respectively), and HC was administered <1 minute after AA and heparinized saline flush.

### 2.3 Clinical and clinicopathological assessment

Physical examinations were performed at baseline (T‐1h), <5 minutes after LPS infusion completion (T0), hourly to T6, and at T12 and T24. Before entering the stall at each examination time point, a pain score was obtained.^49^ As part of each examination, 3 indirect MAP measurements were taken (Passport 8, Mindray, Mahwah, New Jersey) and the mean of the 3 measurements was recorded. Blood was collected from the IV catheter for WBC analysis at T‐1, 1, 2, 6, 12, and 24h, and for SBA at T‐1, 1, 12, and 24h. Whole blood anticoagulated with EDTA and serum were collected at T‐1, 1, 2, 3, 6, and 12h for SAA concentration (StableLab, Zoetis, Parsippany, New Jersey) and serum cortisol concentration, respectively. Serum cortisol concentrations were measured to assess for the presence of CIRCI. All T1h blood samples were collected 2 to 5 minutes before drug administration. A low‐dose ACTH stimulation test (cosyntropin, Sandoz, Inc., Princeton, New Jersey) was performed on horses in the placebo and AA only groups at T1h (<1 minute before drug administration) as an additional test to detect the presence of CIRCI, as determined by a blunted cortisol response to ACTH.^54^ Delta cortisol was calculated as serum cortisol concentration at T2h (60 minutes after ACTH administration) minus serum cortisol concentration at T1h (<1 minute before ACTH administration). Given that HC administration interferes with serum cortisol measurements, ACTH stimulation tests were not performed in horses administered HC.^52^

### 2.4 Inflammatory cytokine analyses

Whole blood and plasma were collected from each horse at T‐1, 1, 2, 3, 6, and 12h to determine pro‐inflammatory cytokine gene expression and plasma concentration, respectively. The cytokines evaluated included TNF‐α, IL‐1β, and IL‐6. For gene expression analysis, whole blood was placed into RNA stabilization tubes within 15 seconds of collection and frozen at −20°C until analysis. RNA was isolated using a benchtop purification instrument (KingFisher Flex System, ThermoFisher Scientific, Inc., Waltham, Massachusetts) and nucleic acid purification kit (MagMAX CORE Nucleic Acid Purification Kit, Applied Biosystems, Beverly, Massachusetts) per manufacturer recommendations, except there was no DNase step and the pelleted RNA was resuspended in 600 μL viral lysis buffer (Invitrogen, ThermoFisher Scientific, Inc.). Gene expression was determined using the relative quantitation method,^55^ where averaged results from all resting blood samples were used as the calibrator for each gene. Beta‐glucuronidase (β‐GUS) was used as the housekeeping gene for all samples,^56^ and samples were assayed using commercially available primers and probes (ThermoFisher Scientific, Inc.)^57^:

• β‐GUS (Ec03470630_m1)

• TNF‐α (Ec03467871_m1)

• IL‐1β (Ec04260298_s1)

• IL‐6 (Ec03468678_m1)

Plasma was separated within 1 hour of collection and stored at −80°C until analysis. Concentrations of TNF‐α, IL‐1β, and IL‐6 were measured from each sample using commercially available enzyme‐linked immunosorbent assay (ELISA) kits validated for use in horses (Horse Elisa Kits, R&D Systems, Minneapolis, Minnesota).^58^, ^59^, ^60^ The mean of duplicate samples was recorded.

### 2.5 Plasma AA concentrations

Plasma AA concentrations were measured at T‐1, 1, 6, and 12h by high‐performance liquid chromatography (HPLC) and mass spectrometry (MS), as described.^61^, ^62^ Plasma was separated within 1 hour of collection and 100 μL was transferred to microcentrifuge tubes containing 400 μL of an aqueous stability solution (6% metaphosphoric acid and 0.25% dithiothreitol) and 10 ng of an internal control (l‐ascorbic acid‐^13^C_6_). The mixture was vortexed and stored at −20°C until analysis. Before the analysis, the mixture was thawed and centrifuged at 16 000*g* for 10 minutes. Then, 100 μL of supernatant was transferred to a new vial and mixed 1 : 1 (vol/vol) with acetonitrile. The HPLC column used was an Intrada Amino Acid 150 mm × 2 mm × 3 μm (Imtakt USA, Portland, Oregon). The binary pump flow rate was set at 0.3 mL/min. Mobile phase A was acetonitrile with 0.3% formic acid and mobile phase B was 20 : 80 (vol/vol) acetonitrile : 100 mM ammonium formate in water. Ten microliters of the reconstituted sample was delivered to the column through an Agilent 1290 Infinity II LC with multisampler (Agilent Technologies, San Jose, California) and a 6470 triple quadrupole mass spectrometer (Agilent Technologies) equipped with Jet Stream ESI ion source. The binary pump used a linear gradient to 80% B in 6 minutes and held for 1 minute, then re‐equilibrated to 100% A in 1 minute followed by a 5 minutes hold. The monitored m/z were 175.0 ➔115.0 and 181.0 ➔119.0 for the endogenous and the internal standard, respectively, and the collision energy was 15 V. Data acquisition and processing utilized Agilent's MassHunter (B.06.00). The peak ratio of l‐ascorbic acid to the internal standard was calculated. Concentrations, reported in ng/mL, were obtained by multiplying the peak ratio by the internal standard amount (10 ng) and dividing by the plasma volume (100 μL). The mean of the duplicate was recorded. To validate this method, linearity and accuracy were tested in duplicate and found to be good (linearity: *R* ^2^ = 0.9997; accuracy: 88.4%).

### 2.6 Statistical analysis

Continuous data were assessed for normality using a Shapiro‐Wilk test. Data for gene expression (polymerase chain reaction [PCR] were log‐transformed to achieve normality. Differences between groups for continuous outcome variables were assessed via a generalized linear mixed model which included the AA‐HC interaction term, with horse as a random effect and treatment groups and time as fixed effects. Differences in least squares means were assessed after the Bonferroni method of adjustment for multiple comparisons. A *P* value <.05 was considered statistically significant for type III fixed effect variables and for Bonferroni‐adjusted *P* values of least squares mean differences. Summary statistics for normally distributed data are presented as mean ± SD.

## 3 RESULTS

**FIGURE 2:**
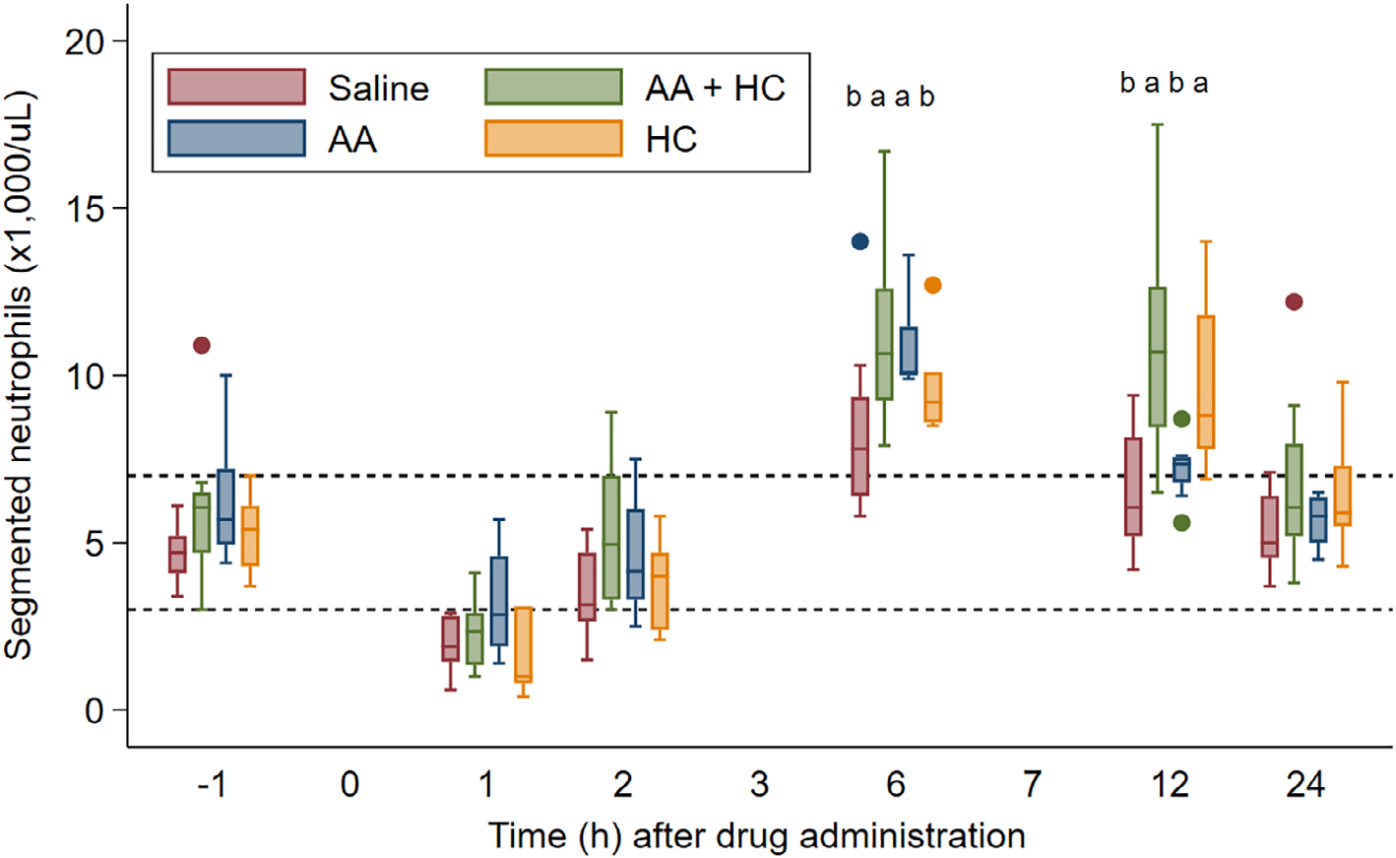
Segmented neutrophil count over time in horses administered 0.9% sodium chloride (placebo; pink box), AA and HC (green box), AA only (blue box), and HC only (yellow box). Within each box, the median is represented by a horizontal line, and brackets represent interquartile range (IQR). Closed circles are outliers, which represent data points outside the IQR with a value >1.5*IQR. Significance between groups is denoted by “a” and “b.” AA, ascorbic acid; HC, hydrocortisone

**FIGURE 3:**
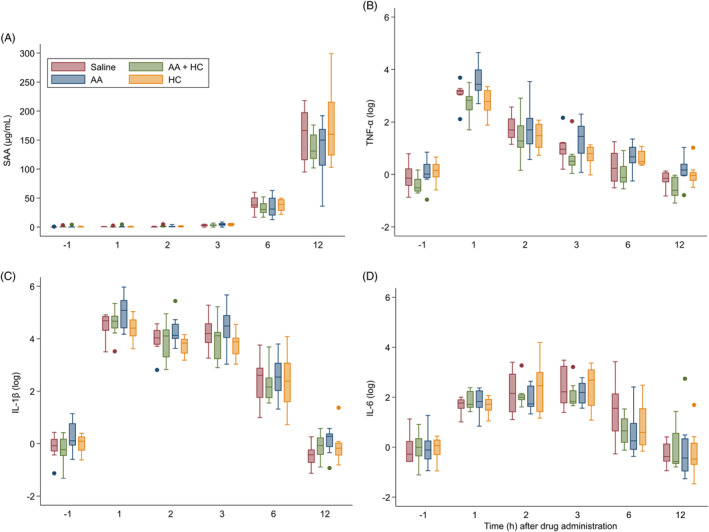
Serum amyloid A (μg/mL), median log TNF‐α plasma concentration, median log IL‐1β plasma concentration, and median log IL‐6 plasma concentration in all horses (n = 32) over time. Within each box, the median is represented by a horizontal line, and brackets represent interquartile range (IQR). Closed circles are outliers, which represent data points outside the IQR with a value >1.5*IQR. IL‐1β, interleukin 1 beta; IL‐6, interleukin 6; SAA, serum amyloid A; TNF‐α, tumor necrosis factor alpha

Lipopolysaccharide infusion resulted in fever, tachycardia, or increased pain score in all horses within 1 hour of completion of the 30‐minute infusion. However, there was no difference in clinical variables among groups at any time point, including rectal temperature, heart rate, pain score, or MAP. As expected, neutropenia was evident within 2 hours of LPS infusion in all horses that received saline (placebo). Six of 8 horses (75%) in the AA + HC group and the HC only group developed neutropenia, while 4 horses (50%) in the AA only group became neutropenic. These differences in neutropenia were not significant among groups (*P* = .18). The segmented neutrophil count was different among treatment groups (*P* = .05). At T6h, horses in the AA + HC and AA only groups had higher segmented neutrophil counts (11.01 ± 1.02 K/µl) than the placebo and HC only groups (8.99 ± 0.94 K/µl; *P* < .009; Figure 2). At T12h, horses in the AA + HC and HC only groupshad higher segmented neutrophil counts (10.4 ± 0.75 K/µl) than horses in the placebo and AA only groups (6.88 ± 0.68 K/µl; *P* < .001; Figure 2). There were no differences in SBA variables among treatment groups at any time point. Serum amyloid A increased over time in all treatment groups (Figure 3), with no difference among groups at any time point (*P* = .58).

### 3.1 Assessment of CIRCI

Intravenous infusion of LPS increased serum cortisol concentration from baseline (T‐1h) to T1h in 29 of 30 horses by a mean ± SD of 99.9% ± 54% (4.53 ± 2.03 μg/dL); 2 horses were not included due to laboratory error. An ACTH stimulation test was performed in horses in the placebo and AA only groups. The mean ± SD delta cortisol concentration after ACTH stimulation in all 16 horses tested (placebo and AA only) was 2.61 ± 1.03 μg/dL, which is less than half of the normal cortisol response in healthy adult horses.^54^ However, there was no difference in delta cortisol concentration between the 2 groups (*P* = .59). Serum cortisol concentrations were increased in the AA + HC and HC only groups compared to other groups at T2 and T12 h (*P* < .003 and *P* < .001, respectively), which corresponded to recent HC administration.

### 3.2 Pro‐inflammatory cytokine analysis

Lipopolysaccharide infusion transiently increased SAA concentrations, pro‐inflammatory cytokine gene expression and pro‐inflammatory cytokine plasma concentrations in all horses (Figure 3). There was no difference in *TNF‐α*, *IL‐1β*, or *IL‐6* gene expression among treatment groups at any time point (*P* = .09, *P* = .14, and *P* = .76, respectively). Similarly, there was no difference in plasma TNF‐α, IL‐1β, or IL‐6 concentration among treatment groups at any time point (*P* ≥ .11).

### 3.3 Plasma AA concentrations

There was a wide range of baseline (T‐1h) plasma AA concentrations among horses (472‐2386 ng/mL). Two horses were removed from analysis due to laboratory errors. Twenty‐five of 30 horses (83%) had a reduction in endogenous plasma AA concentration from baseline to T1h, while 5 of 30 horses (17%) had an increase in plasma AA concentration. Further analysis of endogenous plasma AA concentration in horses that did not receive exogenous AA (placebo and HC only groups) over time to T12h after LPS infusion revealed no difference over time (*P* = .06). Horses that received exogenous AA had increases in plasma AA concentrations at all time points after treatment.

Given the wide range of baseline values, the percent change in plasma AA concentration from baseline to T1h was calculated to further assess the effect of IV LPS infusion on endogenous plasma AA concentrations. The mean ± SD of percent reduction in the 25 horses in which plasma AA concentrations decreased at T1h was 20.1% ± 17.6%, and the mean ± SD of percent increase in the 5 horses in which plasma AA concentrations increased at T1h was 15.9% ± 23.8%. There was no significant difference in percent reduction in plasma AA concentration from baseline to T1h among the 30 horses for which data were available (*P* = .2).

## 4 DISCUSSION

There was no effect of AA, HC, or a combination of AA and HC on clinical signs, plasma SAA concentration, pro‐inflammatory cytokine gene expression, or plasma pro‐inflammatory cytokine concentration compared to placebo controls at any time point. However, administration of AA was associated with higher segmented neutrophil counts 6 and 12 hours after LPS infusion. Development of neutropenia within 2 hours of IV LPS administration is due to margination of mature neutrophils, which is caused by upregulation of adhesion molecules.^63^ Rebound neutrophilia associated with demargination is then observed 6 to 12 hours after LPS exposure after activation of the inflammatory cascade and signaling through IL‐8, IL‐23, and TGF‐β.^64^ In the current study, rebound neutrophilia was more profound for up to 12 hours after LPS exposure in horses that were administered AA. This might be due to mitigation of neutrophil depletion associated with oxidative stress. The interaction of neutrophils with LPS causes immediate release of ROS, and the neutrophils are then destroyed after degranulation.^65^

Excessive ROS release can damage host cells and tissues, contributing to development of organ failure.^65^, ^66^ Ascorbic acid inhibits iNOS expression and ROS production in humans and rodents exposed to LPS, and the same effects are expected in horses.^67^, ^68^, ^69^ Since ROS production was not evaluated in the current study, these possible explanations remain speculative. In addition, anti‐inflammatory effects of AA might decrease LPS‐induced monocyte/macrophage activation, thus decreasing adhesion molecule activation and subsequent neutrophil margination.^63^

Lipopolysaccharide administration IV triggered an increase in plasma SAA concentration in all horses, but there was no effect of treatment at any time point. This major acute phase protein is a reliable marker of infectious and noninfectious inflammation, and can increase >1000‐fold within 6 hours of inflammatory insult.^47^, ^70^, ^71^, ^72^ The mean increase in plasma SAA among horses was relatively mild compared to clinical cases of sepsis, but it is possible that SAA concentrations peaked after the final sampling time point .^71^ This might be due to the low and short‐acting dose of LPS that was administered. It is also possible that later time points including 24 and 48 hours after LPS infusion and treatment might have been needed to detect a difference among treatment groups.

The mean ± SD serum cortisol concentration nearly doubled from baseline to 1 hour after LPS infusion. This is consistent with previous reports that have documented the immediate increase in serum cortisol concentration after induction of experimental endotoxemia in horses.^73^, ^74^ Illness induced by this LPS infusion model appears to have induced CIRCI, as evidenced by a blunted cortisol response to ACTH stimulation 1 hour after stimulation in all horses tested. A study investigating the cortisol response to varying doses of exogenous ACTH in healthy adult horses found that cosyntropin at 0.1 μg/kg, which was the dose used in the current study, increased cortisol 1 hour after stimulation by a mean ± SD of 5.65 ± 1.63 μg/dL.^54^ Given that all horses in the current study were administered LPS, a comparison to normal horses could not be made. However, a blunted cortisol response to ACTH has been defined as a delta cortisol concentration less than the mean delta cortisol concentration achieved in healthy, age‐matched controls using the same ACTH stimulation protocol.^19^ Using this definition, the extent of increase in serum cortisol concentration after ACTH stimulation in the current study (mean ± SD: 2.61 ± 1.03 μg/dL) was less than half of that in normal horses, which is consistent with CIRCI. Furthermore, CIRCI has been documented in adult horses with SIRS, with diagnosis based on increased ACTH : cortisol ratios rather than delta cortisol.^23^ Treatment with AA did not improve the cortisol response compared to placebo controls, suggesting that either the dose of AA was inadequate to decrease illness severity or that AA is ineffective in mitigating CIRCI in horses. The effect of HC treatment was not assessed because of interference in serum cortisol testing, but the likelihood of CIRCI in septic horses suggests that physiologic doses of HC should be further investigated in clinical cases.

In human sepsis, circulating concentrations of AA are markedly depleted.^34^, ^35^, ^65^ Although there was no difference in percent reduction in plasma AA concentration from baseline to T1h among horses, the majority (83%) demonstrated a decrease. This might not have reached significance because of the small sample size, as well as the large variation in both baseline AA concentrations and percent change in AA concentrations among individual horses. Similar variations in endogenous AA concentrations occur in several species, including humans, dogs, cats, and horses.^75^, ^76^, ^77^, ^78^ This might be due to variation in diet and might also reflect differential AA absorption and excretion based on age, sex, and genetic differences.^76^, ^79^, ^80^, ^81^ Variation in peak plasma AA concentrations after parenteral administration occurs in humans, dogs, and horses.^51^, ^82^, ^83^, ^84^ Since none of the horses in the placebo and HC only groups were administered exogenous AA, and HC alone was not expected to mitigate SIRS to an appreciable extent, it was expected that all horses in both groups would demonstrate a decrease in plasma AA concentration over time to T12h, but no difference was found. This might be due to the relatively mild degree of illness induced by the low‐dose of LPS administered. Taken together, it is likely that supplementation with exogenous AA is beneficial in mitigating AA depletion during sepsis, and although pharmacokinetic analysis was not performed here, plasma AA concentrations increased incrementally with IV administration.

There are several limitations to the study. The low dose of LPS used to induce endotoxemia might not have been sufficient to test the potential benefit of AA and HC. However, higher doses of LPS are associated with disease that can be too severe to treat and is ethically challenging.^85^, ^86^, ^87^ In addition, variability in response to LPS among individual horses in combination with a relatively low sample size might have precluded accurate assessment of drug efficacy. Endotoxin tolerance is defined as a diminished response to endotoxin after previous exposure, and has been observed in vivo.^88^, ^89^ Although horses were only included in the study if a clinical response to endotoxemia was observed, the effect of endotoxin tolerance on outcome measures is unknown. Evaluation of drug efficacy in adult horses and neonatal foals with naturally occurring sepsis is ideal, but variability among clinical presentations makes this impractical for proof‐of‐concept studies. Finally, evaluation of oxidative stress markers might have elucidated beneficial effects of AA that were not apparent from the variables assessed here.

The dose of AA that was used in our study was based on pharmacokinetic data in horses and human sepsis studies,^45^, ^51^ but was likely too low to exert substantial anti‐inflammatory and antioxidant effects. The requirement for AA increases with the severity of infection in human patients, and high doses (up to 75 mg/kg q6h IV) continue to be investigated for viral and bacterial human sepsis.^90^, ^91^ In addition, effects of AA have been investigated in human burn patients, with high doses (66 mg/[kg h] for 24 hours) associated with a significant reduction in colloid fluid requirements compared to lower doses.^92^ Taken together, it is possible that higher doses of AA in endotoxic horses could elicit stronger anti‐inflammatory and antioxidant effects, as well as improve the cortisol response to illness.

## CONFLICT OF INTEREST DECLARATION

Dr George E. Moore serves as Consulting Editor for Experimental Design and Statistics for the *Journal of Veterinary Internal Medicine*. He was not involved in review of this manuscript. No other authors have a conflict of interest.

## OFF‐LABEL ANTIMICROBIAL DECLARATION

Authors declare no off‐label use of antimicrobials.

## INSTITUTIONAL ANIMAL CARE AND USE COMMITTEE (IACUC) OR OTHER APPROVAL DECLARATION

This study was approved by Purdue University's IACUC.

## HUMAN ETHICS APPROVAL DECLARATION

Authors declare human ethics approval was not needed for this study.
